# Longitudinal maturation of auditory cortical function during adolescence

**DOI:** 10.3389/fnhum.2015.00530

**Published:** 2015-10-20

**Authors:** Ahren B. Fitzroy, Jennifer Krizman, Adam Tierney, Manto Agouridou, Nina Kraus

**Affiliations:** ^1^Department of Communication Sciences and Disorders, Northwestern UniversityEvanston, IL, USA; ^2^Auditory Neuroscience Laboratory, Northwestern UniversityEvanston, IL, USA; ^3^Institute for Neuroscience, Northwestern UniversityEvanston, IL, USA; ^4^Department of Neurobiology and Physiology, Northwestern UniversityEvanston, IL, USA; ^5^Department of Otolaryngology, Northwestern UniversityEvanston, IL, USA

**Keywords:** CAEP, auditory, cortical, development, adolescence, electrophysiology, variability, longitudinal

## Abstract

Cross-sectional studies have demonstrated that the cortical auditory evoked potential (CAEP) changes substantially in amplitude and latency from childhood to adulthood, suggesting that these aspects of the CAEP continue to mature through adolescence. However, no study to date has longitudinally followed maturation of these CAEP measures through this developmental period. Additionally, no study has examined the trial-to-trial variability of the CAEP during adolescence. Therefore, we longitudinally tracked changes in the latency, amplitude, and variability of the P1, N1, P2, and N2 components of the CAEP in 68 adolescents from age 14 years to age 17 years. Latency decreased for N1 and N2, and did not change for P1 or P2. Amplitude decreased for P1 and N2, increased for N1, and did not change for P2. Variability decreased with age for all CAEP components. These findings provide longitudinal support for the view that the human auditory system continues to mature through adolescence. Continued auditory system maturation through adolescence suggests that CAEP neural generators remain plastic during this age range and potentially amenable to experience-based enhancement or deprivation.

## Introduction

Adolescence is a time of substantial change in brain structure and function. While the bulk of neural cytoarchitectural change occurs shortly after birth (Moore and Guan, 2001; Eggermont and Moore, 2012), decreases in gray matter volume and increases in white matter volume are still evident throughout adolescence (Giedd et al., 1999; Paus et al., 1999; Gogtay et al., 2004; Whitford et al., 2007) suggesting continued refinement of neural circuits via synaptic pruning and increased axonal myelination. This developmental neuroplasticity during adolescence is observed across many brain regions, including the auditory system (Paus et al., 1999; Gogtay et al., 2004). Further, structural changes are accompanied by changes in neural function. In the auditory system, cross-sectional studies have demonstrated changes in the electrophysiological responses to sound across adolescence, indicating that auditory processing in brainstem and cortex continues to mature (Albrecht et al., 2000; Cunningham et al., 2000; Ponton et al., 2000; Bishop et al., 2007; Mahajan and McArthur, 2012; Skoe et al., 2015; Krizman et al., 2015).

**Figure 1:**
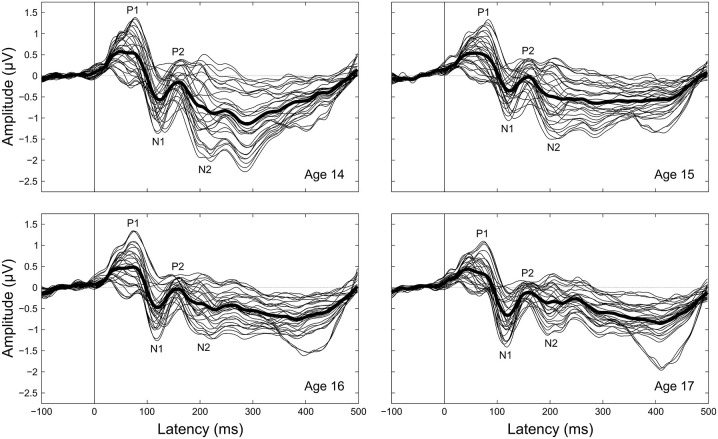
**Grand-averaged CAEPs by age.** Each panel shows the grand averaged waveform at each electrode for a given age, with the thicker black line the average across electrodes. Component labels (P1, N1, P2, N2) are centered on the mean observed latency for each component for each age.

The cortical auditory evoked potential (CAEP), sometimes referred to as the late latency response or obligatory cortical response, is a waveform consisting of positive and negative deflections that occur between 0–300 ms after sound onset (Figure 1). The mature CAEP has four characteristic peaks: a positive deflection centered around 50 ms (P1), a negative deflection centered around 100 ms (N1), a second positive deflection centered around 150 ms (P2), and a second negative deflection centered around 200 ms (N2). These deflections are thought to primarily represent post-synaptic potentials that arise from activity across different neural generators within the auditory system (Vaughan and Ritter, 1970; Scherg and Von Cramon, 1985; Velasco and Velasco, 1986; Näätänen and Picton, 1987; Liégeois-Chauvel et al., 1994; Picton et al., 1999; Ponton et al., 2000; Scarff et al., 2004; Wyss et al., 2014). P1 is thought to originate primarily in the lateral portion of Heschl’s gyrus (Vaughan and Ritter, 1970; Liégeois-Chauvel et al., 1994), and N1 is thought to originate primarily in primary and secondary auditory cortices (Vaughan and Ritter, 1970; Scherg and Von Cramon, 1985; Näätänen and Picton, 1987; Scarff et al., 2004; Wyss et al., 2014). Though the neural origins of P2 and N2 are less well understood, P2 is thought to have contributions from generators in primary and other auditory cortical regions as well as the reticular activating system (Vaughan and Ritter, 1970; Velasco et al., 1985; Velasco and Velasco, 1986; Crowley and Colrain, 2004; Wyss et al., 2014), and N2 is thought to have multiple generators across brainstem, thalamic, and cortical auditory regions (Velasco et al., 1985; Velasco and Velasco, 1986; Ponton et al., 2002; Mahajan and McArthur, 2012).

Cross-sectional studies have demonstrated that the latencies and amplitudes of CAEP components change over the course of adolescence (e.g., Ponton et al., 2000; Bishop et al., 2007; see Mahajan and McArthur, 2012 for detailed review). Studies have repeatedly shown that P1 gets earlier and smaller, N1 gets earlier and larger, and N2 gets smaller during adolescence (Enoki et al., 1993; Johnstone et al., 1996; Oades et al., 1997; Sharma et al., 1997; Cunningham et al., 2000; Pang and Taylor, 2000; Ponton et al., 2000; McArthur and Bishop, 2002; Bishop et al., 2007; Sussman et al., 2008; Mahajan and McArthur, 2012). However, whether P2 amplitude, P2 latency, or N2 latency continue to mature during this age range is more contentious. Multiple studies have reported stable P2 amplitude and/or latency during adolescence (Martin et al., 1988; Johnson, 1989; Johnstone et al., 1996; Tonnquist-Uhlén, 1996; Ponton et al., 2000; Mahajan and McArthur, 2012), but increases and decreases in P2 amplitude and latency have also been observed (Goodin et al., 1978; Martin et al., 1988; Johnstone et al., 1996; Tonnquist-Uhlén, 1996; Oades et al., 1997; Albrecht et al., 2000; Ponton et al., 2000; Sussman et al., 2008; Mahajan and McArthur, 2012). Similarly, multiple studies have reported decreasing N2 latency during adolescence (Martin et al., 1988; Tonnquist-Uhlén, 1996; Cunningham et al., 2000; Sussman et al., 2008), but unchanging, increasing, and nonlinearly changing N2 latencies have also been reported (Goodin et al., 1978; Johnson, 1989; Enoki et al., 1993; Johnstone et al., 1996; Oades et al., 1997; Ponton et al., 2000; Mahajan and McArthur, 2012). A potential explanation for the discrepant P2 and N2 findings is that the cross-sectional studies obscure maturation with individual differences. We reasoned that longitudinally following the development of the CAEP in the same adolescents would clarify these ambiguities.

Additionally, recent evidence demonstrates that within-individual variability of the CAEP is an important metric of age-dependent differences in auditory processing during active listening tasks (Strait and Kraus, 2011; Strait et al., 2014). Specifically, an increase in the variability of the CAEP to actively ignored sounds is observed between childhood and adulthood, while the variability of the CAEP to actively attended sounds does not change during this time (Strait et al., 2014). However, it is not yet known how the trial-to-trial variability of CAEPs elicited under passive listening conditions, like those typically employed in clinical assessments of auditory function, changes with age. Passively elicited CAEPs are typically recorded while the participant performs an unrelated activity such as watching a movie (e.g., McArthur and Bishop, 2002; Sussman et al., 2008), so it is possible that the passively elicited CAEP is more comparable to the CAEP elicited by actively ignored sounds than to the CAEP elicited by actively attended sounds. If so, passively elicited CAEP variability should follow the same maturational trajectory as actively ignored CAEP variability and increase during adolescence. Further, studies examining CAEP variability have thus far treated the P1-to-N2 time window as a single entity (Strait and Kraus, 2011; Krizman et al., 2014; Strait et al., 2014). Considering that these components stem from different neural generators and show differences in amplitude and latency maturation, it is reasonable to assume that they may also show differences in response variability maturation; this assumption can be tested by assessing the variability of individual CAEP components separately rather than as a combined group.

To address these issues, we longitudinally tracked the latency, amplitude, and variability of the passively collected CAEP to the speech syllable [da] in a cohort of adolescents from age 14 years to age 17 years. This age range was longitudinally followed because it is a known period of developmental neuroplasticity (Paus, 2005), but cross-sectional comparisons of cortical AEP responses between children and adults have offered inconsistent insights into the neural changes that are taking place during this period. From these previous cross-sectional findings, we predicted that P1 would get earlier and smaller, N1 would get earlier and larger, P2 would not change in timing or amplitude, and N2 would get earlier and smaller during this age range. Additionally, given previous age-dependent findings regarding CAEP variability we sought to inform our understanding of passively-evoked response variability.

## Materials and Methods

### Participants

Sixty-eight adolescents (thirty-nine female) were recruited from three public high schools in the Chicago metropolitan area; the data were collected over 4 years as part of a longitudinal study in the Auditory Neuroscience Laboratory. Participants were enrolled in the study during the summer prior to their first year of high school (age at first test: *M* = 14.63 years, *SD* = 0.42 years) and returned once a year for the next three years (test-retest interval: *M* = 11.22 months, *SD* = 1.82 months). All participants had normal hearing at each session (defined as air conduction thresholds of <20 dB nHL at each octave from 125–8000 Hz), had no diagnosed neurological disorders, and had an IQ ≥80 at the start of the study, as measured by the Wechsler Abbreviated Scale of Intelligence (WASI; Wechsler, 1999). Additional participant demographic information can be found in Table 1. Data were collected from one additional participant but excluded from analyses due to poor electroencephalogram (EEG) data quality in one of the 4 years.

**Table 1:** **Participant demographics**.

			*Maternal education*			
*Sex*	*Handedness*	*Full scale IQ M (SD)*	Middle school	High school	College	Graduate school
29 M, 39 F	65 R, 3 L	98.54 (9.56)	9	20	32	5

*Number of participants separated by sex, handedness, and self-reported maternal education level at the time of study entry are reported. Two participants did not specify maternal education level. Mean and standard deviation two-subtest full scale IQ scores on the WASI (Wechsler, 1999) at the time of study entry are also reported for the entire participant cohort*.

### Stimuli

During the recording session, participants were continuously presented with a synthesized (Klatt, 1980; Parbery-Clark et al., 2009) 170 ms consonant-vowel syllable ([da]) followed by an inter-stimulus-interval of 836 ms (inter-onset-interval of 1006 ms). The [da] stimulus was presented with randomized polarity using NeuroScan Stim^2^ software (Compumedics, Charlotte, NC, USA), with an equal probability of condensation and rarefaction polarity on each stimulus presentation.

### Procedure

All protocols and procedures were approved by the Northwestern University Institutional Review Board. In accordance with the Declaration of Helsinki, parental or guardian written informed consent and adolescent written informed assent were obtained for all participants prior to testing at each data collection session. During the EEG recording session participants sat in a comfortable chair in a darkened room watching a movie of their choice at low volume (<40 dB SPL) in free field while the [da] speech stimulus was presented monaurally to the right ear via insert earphone (ER-3A; Etymōtic Research, Elk Grove Village, IL, USA) at 80 (±1) dBa SPL. Participants’ left ears were unoccluded to hear the movie soundtrack. Participants were informed that they did not need to attend to the sounds and that they could focus on watching the movie.

### Data Collection and Processing

EEG data were collected using a 31-channel electrode (Sn) cap (Compumedics, Charlotte, NC, USA) referenced to the linked earlobes at a sampling rate of 500 Hz and a bandpass of 0.05–100 Hz. Vertical and horizontal electrooculogram (VEOG, HEOG) data were collected using the same parameters with electrodes centered above the left eye and beside the outer canthus of the left eye respectively. EEG data were preliminarily epoched online to monitor for large artifacts (voltage exceeding ±100 μV at any electrode), and recording continued until 400 responses to [da] without large artifacts had been obtained. This online processing was performed solely to ensure sufficient data collection; all processing and analysis steps reported hereafter were performed offline starting from the raw continuous EEG.

Continuous EEG data were filtered offline from 1–35 Hz using a fourth order IIR Butterworth filter with 24 dB/octave rolloff. Filtered EEG data were then segmented into 600 ms epochs beginning 100 ms before sound onset. Individual epochs were baseline corrected to the 100 ms pre-stimulus period during segmentation. Epochs containing eyeblinks (defined as VEOG voltage range exceeding 50 μV within a 150 ms moving window [120 ms increments]), eye movements (defined as step-like HEOG voltage change exceeding 100 μV within a 200 ms moving window [50 ms increments]), or excessive noise (voltage exceeding ±170 μV at any EEG electrode) were automatically detected and excluded from further analyses. Artifact-free epochs were then averaged separately for each channel and participant. EEG data processing was performed in MATLAB (The Mathworks, Inc., Natick, MA, USA) using a combination of EEGLAB (Delorme and Makeig, 2004), ERPLAB (Lopez-Calderon and Luck, 2014), and custom functions written by AF and AT.

### CAEP Analyses

**Figure 2:**
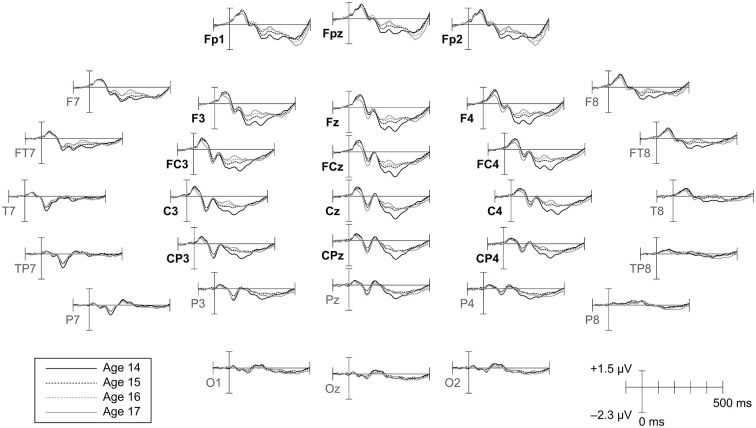
**Topographic distribution of CAEPs by age.** Grand average waveforms for each age are shown at all electrodes. Ages 14 and 17 are plotted as solid lines to highlight changes across the duration of the study. Electrodes included in the frontrocentral region of interest (ROI) employed for amplitude and variability analyses are labeled in bold.

Peak latency, mean amplitude, and variability measurements were taken on four components (P1, N1, P2, N2) clearly evident in the grand averaged CAEP waveforms (Figures 1, 2). To supplement the peak latency and mean amplitude measures of individual components, inter-peak latencies and inter-peak amplitudes were measured between three component pairs (P1/N1, N1/P2, P2/N2).

#### Latency

**Figure 3:**
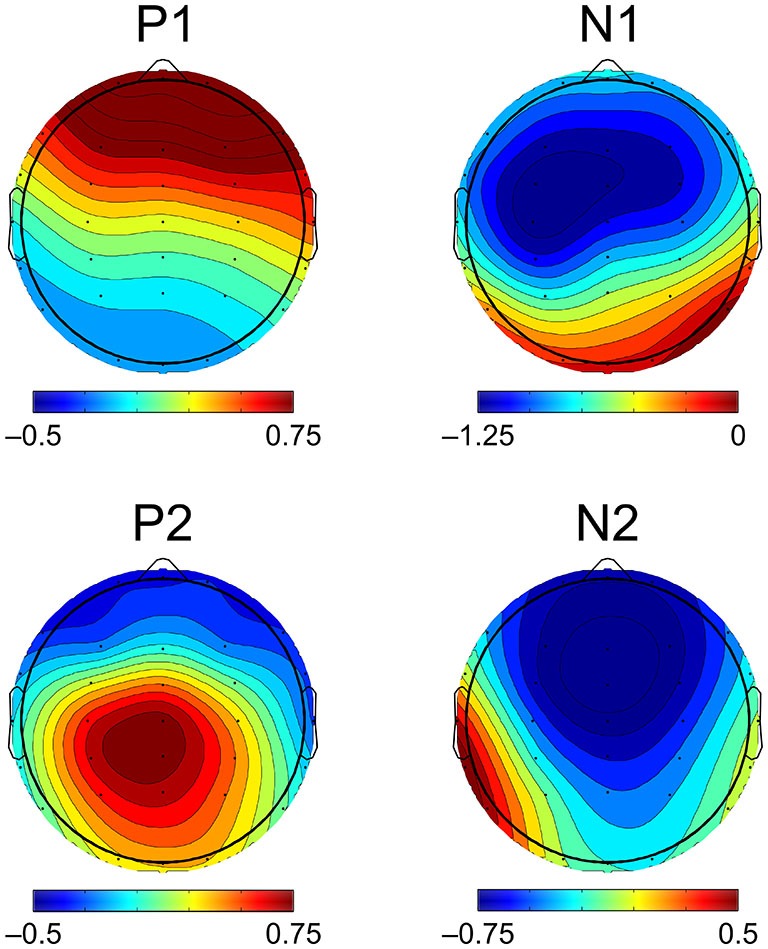
**Topographic distribution of CAEP components.** CAEP component topography is visualized here as the scalp distribution of mean amplitudes of the grand averaged CAEP across all participants and ages. Scalp maps show mean amplitudes measured within the same time window widths used for statistical analyses (P1, N1, P2 = 40 ms, N2 = 70 ms), centered on the peak component latencies of the grand average. N1, P2, and N2 mean amplitudes shown here were measured using the mean amplitude of the preceding component as a baseline, while P1 mean amplitude was measured using the mean amplitude of the prestimulus period as a baseline.

Component peak latencies were manually identified for each participant by experienced researchers (AF, JK, AT) simultaneously viewing: (1) separate overlays of all 31 recording electrodes and the across-electrode average for ages 14 through 17 (similar to Figure 1); (2) topographical waveform plots for ages 14 through 17 (similar to Figure 2); and (3) an overlay of the across-electrode average waveforms for ages 14 through 17 (similar to Figure 6). Based on grand average morphology and topography (Figures 2, 3), P1 was defined as the largest positive or polarity inverting peak between 40–100 ms, N1 was defined as the largest negative peak concentrated over left central scalp regions between 65–160 ms, P2 was defined as the largest positive peak following N1 between 100–220 ms, and N2 was defined as the largest negative peak concentrated over left frontal scalp regions between 140–290 ms. All manually picked latencies were reviewed a second time by a single experienced researcher (AF) simultaneously viewing all 4 years of data to ensure that components were identified consistently within each participant across years. Inter-peak latencies were measured for each component pair (e.g., P1/N1) by subtracting the latency identified for the first component from the latency identified for the second component.

#### Amplitude

**Figure 4:**
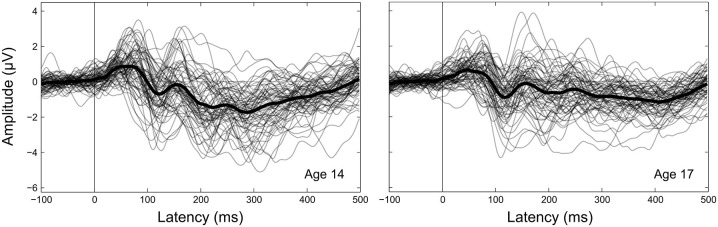
**Between-participant CAEP variability.** CAEPs are shown as individual waveforms for each participant at age 14 (left) and age 17 (right), averaged across the 15 electrodes in the frontocentral ROI. Individual participant CAEPs are plotted with partial transparency to visualize between-participant variability, with darker plot regions indicating lower variability. The grand average CAEP across all participants is shown as a thicker solid black line for each year.

Component amplitudes were measured by taking the mean voltage at each electrode within a fixed-width time window (P1, N1, P2: 40 ms, N2: 70 ms) centered on the component peak latency identified for that participant in that year. These time window widths were determined by identifying 50% amplitude points between peak amplitudes of the grand averaged waveforms and measuring durations between them. A latency-centered approach was employed due to the wide variety of component timing observed across participants, especially at age 14 (Figure 4). For any participant who did not have an identifiable component peak in a given year, the mean amplitude measurement window was instead centered on the mean peak latency observed for that component across all participants with an identifiable peak during that year. This approach ensured that participants with very small component amplitudes were included in analyses, increasing the meaningful range over which the mean amplitude measurements were distributed. Inter-peak amplitudes were measured for each component pair (e.g., P1/N1) by subtracting at each electrode the instantaneous amplitude measured at the peak latency of the second component from the instantaneous amplitude measured at the peak latency of the first component. Because the observed CAEP waveforms were predominantly distributed over frontal and central scalp regions across all 4 years (Figures 2, 3), mean and inter-peak amplitude measures were averaged across electrodes within a frontocentral region of interest (ROI) prior to statistical analyses. The frontocentral ROI contained 15 electrodes (Figure 2): Fp1, Fpz, Fp2, F3, Fz, F4, FC3, FCz, FC4, C3, Cz, C4, CP3, CPz, CP4.

#### Variability

**Figure 5:**
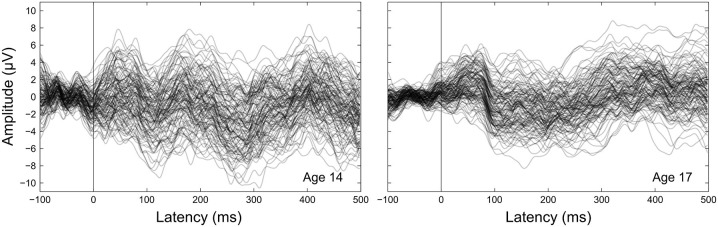
**Within-participant CAEP variability.** Panels show subaverages from 25 iterations of the bootstrapping procedure used to calculate CAEP variability from the same individual at age 14 (left) and age 17 (right). Each iteration randomly divides a fixed population of 150 artifact-free epochs into five 30-epoch subaverages, across which the CAEP variability metrics are calculated. Final CAEP variability measures are the averaged results of 1000 iterations of this procedure. Subaverages are plotted with partial transparency to visualize within-participant variability, with darker plot regions indicating lower variability.

Component variabilities were measured using a bootstrapping procedure to quantify trial-to-trial variability within the same fixed-width (P1, N1, P2: 40 ms, N2: 70 ms), peak latency-centered time windows used for the mean amplitude measurements. All variability calculations were performed on a composite electrode created by averaging EEG data across the 15 electrodes in the frontocentral ROI (Figure 2). For each participant and component, 150 artifact-free epochs were randomly sampled without replacement from the set of all artifact-free epochs collected for that participant. For each epoch in this fixed population, the mean voltage within the component time window of interest was standardized to zero by subtracting the mean from each time point. On each bootstrapping iteration (*N* = 1000), five subaverages consisting of 30 epochs each were randomly sampled without replacement from the fixed population of 150 epochs (Figure 5). The voltage standard deviation across the five subaverages was then calculated at each time point in the component time window, and these standard deviations were averaged across the time window to give an overall metric of component variability. The resulting variability metrics from each of the 1000 bootstrapping iterations were averaged to give the final component variability measures.

### Statistical Analyses

For each dependent variable a one-way repeated-measures ANOVA with age (14, 15, 16, 17) as the within-subjects factor was performed. When a significant or trending main effect of age was observed in the ANOVA, ages 14 and 17 were directly compared using a two-tailed paired-samples *t*-test. If a main effect was observed in the ANOVA but no differences were observed between ages 14 and 17, follow-up two-tailed paired-samples *t-tests* were conducted to test shorter maturational trends evident in the means across ages. Additionally, similar variability results across components motivated a *post hoc* omnibus ANOVA adding time window as a factor with levels P1, N1, P2, N2, and the prestimulus period (−100–0 ms) to determine the component specificity of the observed effects. When Mauchly’s test revealed violations of the sphericity assumption of an ANOVA, Hyunh-Feldt corrected degrees of freedom were used to calculate ANOVA *p*-values. To reduce the likelihood of Type I error due to multiple comparisons, the significance of *post hoc* *t-tests* was assessed using Holm-Bonferroni adjusted *p*-values. Participants who did not have identifiable peaks for a component across all ages were excluded from analyses of peak-derived measures (latency, inter-peak latency, and inter-peak amplitude) for that component, because these measures cannot be validly assessed when no peak is evident. However, because the lack of a clear component peak indicates very small component amplitude and therefore adds meaningful information to analyses across individuals, these participants were included in analyses of timewindow-derived measures (mean amplitude and variability) that do not rely on clear peaks for measurement. Peak detectability rates are reported for each component in each year and across all years in Table 2. Statistical analyses were performed in R (R Core Team, 2014; R Studio Team, 2014) using packages reshape2 (Wickham, 2007), ez (Lawrence, 2013), and custom functions coded by AF.

**Table 2:** **Peak detectability**.

Component	Age 14	Age 15	Age 16	Age 17	All years
P1	60 (88.2%)	58 (85.3%)	60 (88.2%)	65 (95.6%)	50 (73.5%)
N1	66 (97.1%)	65 (95.6%)	67 (98.5%)	67 (98.5%)	63 (92.6%)
P2	63 (92.6%)	58 (85.3%)	62 (91.2%)	61 (89.7%)	53 (77.9%)
N2	65 (95.6%)	58 (85.3%)	60 (88.2%)	60 (88.2%)	48 (70.6%)

*Number of participants for whom component peaks were identifiable in each individual year and across all years. The percent of the total number of participants (*n* = 68) represented is expressed in parentheses*.

## Results

### Latency

**Figure 6:**
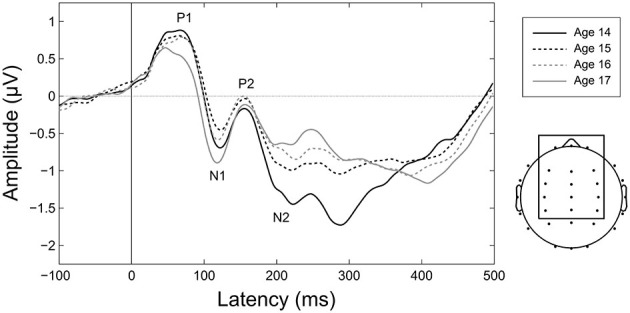
**Maturation of CAEPs during adolescence.** Grand averages for each age over the 15 electrodes in the frontocentral ROI employed for amplitude and variability analyses (shown at right). Ages 14 and 17 are plotted as solid lines to highlight changes across the duration of the study. Component labels (P1, N1, P2, N2) are centered on the mean observed peak latency for each component across all years.

**Figure 7:**
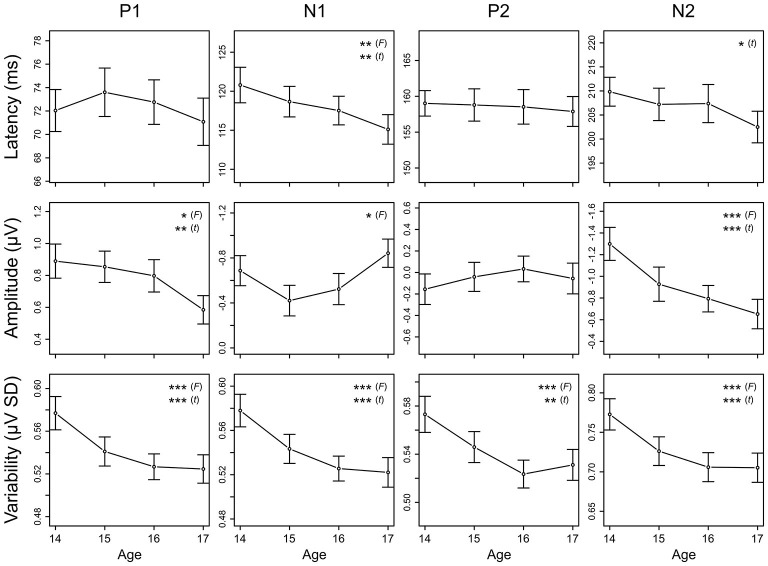
**Longitudinal trajectories of CAEP measures.** Mean and standard error values are plotted across all ages for peak latency, mean amplitude, and variability of P1, N1, P2, and N2. Measures for which the ANOVA *F*-test across all ages or the *t*-test between ages 14 and 17 showed significant maturation effects are indicated by asterisks (* = *p* < 0.05, ** = *p* < 0.01, *** = *p* < 0.001). These data are also reported in Tables 3, 4, and 5. Negative is plotted up for N1 and N2 mean amplitude so that up consistently represents increased magnitude across all plots.

N1 and N2 latencies decreased across adolescence, whereas P1 and P2 latencies remained stable (Figures 6, 7; Table 3). N1 peak latency decreased with age (*F*_(3, 186)_ = 4.596, *p* = 0.004; *t*_(62)_ = 3.615, *p* = 0.001); this effect was further evident as a decrease in P1/N1 inter-peak latency (*t*_(48)_ = 2.726, *p* = 0.009) and an increase in N1/P2 inter-peak latency (*t*_(51)_ = −2.185, *p* = 0.033) from age 14 to age 17. N2 peak latency decreased from age 14 to age 17, (*t*_(47)_ = 2.325, *p* = 0.024; Figures 6, 7), though the main effect of age on N2 latency did not reach significance (*p* < 0.1). This effect was further evident as a decrease in P2/N2 inter-peak latency across all years (*F*_(3, 129)_ = 2.629, *p* = 0.060; *t*_(43)_ = 2.390, *p* = 0.021). Neither P1 nor P2 latency changed with age (*p*’s > 0.4).

**Table 3:** **Latency**.

Peak latency (ms)							
	Mean (SD)			
Component	Age 14	Age 15	Age 16	Age 17	*n*	ME of age (*p*)	14/17 diff (*p*)
P1	72.04 (12.67)	73.60 (14.62)	72.76 (13.45)	71.08 (14.28)	50	0.441	–
N1	120.79 (18.03)	118.67 (15.51)	117.52 (14.53)	115.11 (15.05)	63	**0.004	**0.001
P2	159.02 (12.94)	158.79 (16.40)	158.53 (17.54)	157.89 (15.17)	53	0.950	–
N2	209.83 (20.78)	207.21 (23.29)	207.38 (27.44)	202.50 (22.70)	48	0.112	*0.024

Mean component peak latencies are shown for each age, with standard deviations in parentheses. The number of participants included in statistical comparisons for each measure is shown, along with the *p*-value for the main effect of age in the repeated measures ANOVA and if warranted the *p*-value for the two-tailed paired-samples *t*-test between ages 14 and 17. Significant results are indicated with asterisks (* = *p* < 0.05, ** = *p* < 0.01, *** = *p* < 0.001).

### Amplitude

P1 amplitude decreased, N1 amplitude increased, and N2 amplitude decreased across adolescence, whereas P2 amplitude remained stable (Figures 6, 7; Table 4). P1 mean amplitude decreased with age (*F*_(3, 201)_ = 2.936, *p* = 0.034; *t*_(67)_ = 2.847, *p* = 0.006); this effect was further evident as a decrease in P1/N1 inter-peak amplitude with age (*F*_(3, 144)_ = 3.292, *p* = 0.022; *t*_(48)_ = 3.097, *p* = 0.003). N1 mean amplitude changed with age (*F*_(3, 201)_ = 3.718, *p* = 0.012), remaining stable from age 14 to age 15 (*p* > 0.05) and then increasing from age 15 to age 17 (*t*_(67)_ = 2.914, *p* = 0.005). This trajectory was further reflected in N1/P2 inter-peak amplitude (*F*_(3, 153)_ = 4.753, *p* = 0.004), which also remained stable from age 14 to age 15 (*p* > 0.1) and increased from age 15 to age 17 (*t*_(51)_ = 4.011, *p* = 0.001). N2 mean amplitude decreased with age (*F*_(3, 201)_ = 9.166, *p* < 0.001; *t*_(67)_ = −4.727, *p* < 0.001); this effect was further evident as a decrease in P2/N2 inter-peak amplitude across all years (*F*_(3, 129)_ = 6.792, *p* < 0.001; *t*_(43)_ = 4.210, *p* < 0.001). P2 amplitude did not change with age (*p* > 0.5).

**Table 4:** **Amplitude**.

Mean amplitude (μV)							
	Mean (SD)			
Component	Age 14	Age 15	Age 16	Age 17	*n*	ME of age (*p*)	14/17 diff (*p*)
P1	0.89 (0.88)	0.85 (0.81)	0.80 (0.84)	0.58 (0.73)	68	*0.034	**0.006
N1	−0.69 (1.10)	−0.42 (1.12)	−0.52 (1.14)	−0.84 (1.04)	68	*0.012	0.256
P2	−0.16 (1.17)	−0.04 (1.12)	0.03 (0.99)	−0.06 (1.18)	68	0.578	–
N2	−1.30 (1.25)	−0.93 (1.30)	−0.79 (1.01)	−0.65 (1.12)	68	***<0.001	***<0.001

**Inter-peak amplitude (μV)**							

	**Mean (SD)**			
**Component pair**	**Age 14**	**Age 15**	**Age 16**	**Age 17**	***n***	**ME of age (*p*)**	**14/17 diff (*p*)**

P1/N1	2.54 (1.22)	2.07 (1.08)	2.19 (1.20)	2.09 (0.97)	49	*0.022	**<0.003
N1/P2	−1.24 (1.37)	−0.99 (1.00)	−1.27 (1.09)	−1.48 (1.22)	52	**0.005	0.113
P2/N2	2.15 (1.42)	1.73 (1.26)	1.64 (1.12)	1.43 (1.02)	44	***<0.001	***<0.001

Mean component amplitudes and inter-peak amplitudes are shown for each age, with standard deviations included in parentheses. The number of participants included in statistical comparisons for each measure is shown, along with the *p*-value for the main effect of age in the repeated measures ANOVA and if warranted the *p*-value for the two-tailed paired-samples *t*-test between ages 14 and 17. Significant results are indicated with asterisks (* = *p* < 0.05, ** = *p* < 0.01, *** = *p* < 0.001).

### Variability

Variability decreased for all CAEP components across adolescence (Figure 7; Table 5). This decrease was observed separately for P1 (*F*_(3, 201)_ = 9.732, *p* < 0.001; *t*_(67)_ = 4.188, *p* < 0.001), N1 (*F*_(3, 201)_ = 11.606, *p* < 0.001; *t*_(67)_ = 4.773, *p* < 0.001), P2 (*F*_(3, 201)_ = 9.387, *p* < 0.001; *t*_(67)_ = 3.561, *p* = 0.001), and N2 (*F*_(3, 201)_ = 10.860, *p* < 0.001; *t*_(67)_ = 4.586, *p* < 0.001). *Post hoc* analyses demonstrated that the decrease in variability was evident also during the prestimulus period (*F*_(3, 201)_ = 7.560, *p* < 0.001; *t*_(67)_ = 3.996, *p* < 0.001), and that the observed decreases in variability were not different across components (prestimulus, P1, N1, P2, N2; *p* > 0.2).

**Table 5:** **Variability**.

Variability (within-participant μV SD)							
	Mean (SD)			
Component	Age 14	Age 15	Age 16	Age 17	*n*	ME of age (*p*)	14/17 diff (*p*)
P1	0.58 (0.13)	0.54 (0.11)	0.53 (0.10)	0.52 (0.11)	68	***<0.001	***<0.001
N1	0.58 (0.12)	0.54 (0.11)	0.53 (0.09)	0.52 (0.10)	68	***<0.001	***<0.001
P2	0.57 (0.12)	0.55 (0.11)	0.52 (0.10)	0.53 (0.11)	68	***<0.001	***<0.001
N2	0.77 (0.16)	0.73 (0.15)	0.71 (0.15)	0.71 (0.15)	68	***<0.001	***<0.001
Prestimulus	0.87 (0.20)	0.82 (0.16)	0.81 (0.17)	0.80 (0.16)	68	***<0.001	***<0.001

Mean component variabilities are shown for each age, with standard deviations included in parentheses. The number of participants included in statistical comparisons for each measure is shown, along with the *p*-value for the main effect of age in the repeated measures ANOVA and the *p*-value for the two-tailed paired-samples *t*-test between ages 14 and 17. Significant results are indicated with asterisks (* = *p* < 0.05, ** = *p* < 0.01, *** = *p* < 0.001).

## Discussion

The current study longitudinally demonstrates that cortical auditory circuits continue to mature during adolescence, and that the neural circuits underlying different components of the CAEP (P1, N1, P2, N2) mature in different ways during this developmental period. Specifically, we observed a smaller P1, an earlier and increasing N1, stable P2 timing and amplitude, and an earlier and smaller N2 across this age range. These findings are broadly consistent with prior findings from cross-sectional research (e.g., Ponton et al., 2000; Bishop et al., 2007; Mahajan and McArthur, 2012). Additionally, the present data clearly demonstrate that CAEP variability decreases between the ages of 14 and 17 in a similar manner across all components, suggesting that the passive cortical response to sound becomes more stable during adolescence.

The traditional structural hallmarks of adolescent brain development are increases in cerebral white matter volume (Giedd et al., 1999; Paus et al., 1999; Paus, 2005; Whitford et al., 2007) and decreases in cerebral gray matter volume (Giedd et al., 1999; Gogtay et al., 2004; Whitford et al., 2007). These changes are thought to reflect increases in the myelination of existing white matter fiber tracts (Paus et al., 2001) and reductions in synaptic density as a function of synaptic pruning (Huttenlocher, 1979) respectively. Increases in myelination are associated with increases in neural transmission speed and in turn earlier evoked potential peak latencies (Hecox and Galambos, 1974; Shaw, 1988). Reductions in gray matter volume during adolescence thought to be driven by synaptic pruning are associated with decreases in overall neural activity as evidenced by reduced scalp-recorded EEG power (Whitford et al., 2007). In light of this work, we suggest that the observed reductions in CAEP latency reflect increased neural transmission speed due to increased myelination, that the observed changes in CAEP amplitude reflect greater efficiency in component neural generators due to synaptic pruning, and that the observed reduction in CAEP variability reflects a stabilizing of cortical auditory circuits as a function of these changes during adolescence.

### Maturation of CAEP Latency and Amplitude

#### P1

We observed stable P1 latency across adolescence. This finding was unexpected given previous reports of decreasing P1 latency from childhood to adulthood (Oades et al., 1997; Sharma et al., 1997; Cunningham et al., 2000; Ponton et al., 2000; McArthur and Bishop, 2002; Sussman et al., 2008; Mahajan and McArthur, 2012). Given that these previous studies investigated P1 latency maturation over a much wider age band (ages 5–20+ years), one possible interpretation of this difference is that P1 latency maturation occurs over a sufficiently protracted timescale that no change is evident between the ages of 14 and 17. Alternatively, it may be that P1 latency declines rapidly between age 17 and adulthood. While the general trend across previous studies for P1 latency to decrease from childhood through adulthood suggests that myelination is increasing in auditory white matter pathways that precede activation of P1 generators during adolescence, the lack of clear P1 latency decrease between the ages of 14 and 17 in studies that have examined this age range suggests that this process is either slow or begins in late adolescence.

We observed a reduction in P1 amplitude across adolescence, consistent with previous cross-sectional reports (Oades et al., 1997; Sharma et al., 1997; Cunningham et al., 2000; Ponton et al., 2000; McArthur and Bishop, 2002; Bishop et al., 2007; Sussman et al., 2008; Mahajan and McArthur, 2012). It has been proposed that in normal hearing individuals this reduction in P1 amplitude is at least partially driven by increasing contributions of N1 into the P1 time window as N1 gets larger with age (Ponton and Eggermont, 2001). In this view as N1 becomes larger (more negative), P1 appears to become smaller (more negative). However, we observed a clear decrease in P1 amplitude from age 14–17 concurrent with an increase in N1 amplitude evident only between ages 15 and 17. If the P1 amplitude effect was driven entirely by changes in N1 amplitude we would expect P1 and N1 to follow identical amplitude trajectories across years. Given that they do not, we can conclude that the reduction in P1 amplitude observed in normal hearing individuals during adolescence at least in part reflects maturation of the neural generators of P1, consistent with the findings of Ponton and Eggermont (2001). We suggest that the reduction in P1 amplitude reflects synaptic pruning of the neural circuitry underlying this response, resulting in increased efficiency of P1-generating circuits. To the extent that synaptic pruning is occurring in these circuits, P1 generation will involve fewer neurons due to a decreased spread of activation. This reduced neural activity would reduce the apparent size of P1 at the scalp; our observed reduction in P1 amplitude is consistent with this interpretation.

#### N1

We observed a reduction in N1 latency across adolescence, which is consistent with the majority of previous cross-sectional studies (Johnstone et al., 1996; Oades et al., 1997; Sharma et al., 1997; Albrecht et al., 2000; Cunningham et al., 2000; Ponton et al., 2000; McArthur and Bishop, 2002; Mahajan and McArthur, 2012). Primary auditory cortex is thought to be the predominant generator of N1 (Näätänen and Picton, 1987), but the typical latency of N1 indicates that it is not generated by the first afferent pass through this region (Mäkelä and Hari, 1992; Ponton et al., 2000). Rather, prior to activation of the neurons underlying N1, the auditory signal must travel out of primary auditory cortex to other ipsi- and/or contralateral auditory cortical regions and then back again. Consistent with imaging evidence (Reiss et al., 1996; Paus et al., 1999), the observed reduction in N1 latency therefore suggests that myelination of these cortico-cortical connections has increased during adolescence leading to an increase in transmission speed. Further, the absence of latency change for P1 from 14–17 years old supports the idea that the decrease in N1 latency is driven by myelination of primarily cortico-cortical rather than thalamo-cortical pathways.

We observed a change in N1 amplitude between ages 14 and 17 years, with a clear increase in N1 amplitude between ages 15 and 17 years. While there was no amplitude change between ages 14 and 15 (see Goodin et al., 1978), the eventual increase in N1 amplitude is consistent with previous cross-sectional work (Goodin et al., 1978; Martin et al., 1988; Oades et al., 1997; Cunningham et al., 2000; Pang and Taylor, 2000; Ponton et al., 2000; McArthur and Bishop, 2002; Bishop et al., 2007; Sussman et al., 2008; Mahajan and McArthur, 2012). This increase in N1 amplitude suggests greater neuronal activation occurs during N1 generation with age during adolescence, resulting either from the recruitment of neurons to the N1-generating circuit or the excitation or disinhibition of neurons already in the circuit. Though it is not possible to disambiguate these reorganization mechanisms based on the present data, imaging has shown that gray matter density loss occurs later in parietal regions that support attention than in sensory regions like primary auditory cortex (Posner et al., 1984; Gogtay et al., 2004; Buschman and Miller, 2007). Thus, one possible explanation for the observed increase in N1 amplitude would be synaptic pruning in inhibitory attentional circuits beginning during adolescence; this hypothesis could be tested by future animal work relating parietal synaptic density to N1 amplitude.

#### P2

We observed stable P2 latency and amplitude across adolescence, consistent with multiple previous cross-sectional studies (Martin et al., 1988; Johnson, 1989; Johnstone et al., 1996; Tonnquist-Uhlén, 1996; Ponton et al., 2000; Mahajan and McArthur, 2012). However, it should be noted that cross-sectional studies have also reported changing P2 latency (Goodin et al., 1978; Tonnquist-Uhlén, 1996; Oades et al., 1997; Albrecht et al., 2000; Sussman et al., 2008; Mahajan and McArthur, 2012) or changing P2 amplitude (Goodin et al., 1978; Martin et al., 1988; Johnstone et al., 1996; Oades et al., 1997; Albrecht et al., 2000; Ponton et al., 2000; Sussman et al., 2008) during adolescence. While it is not immediately clear why the results of previous cross-sectional investigations into adolescent P2 maturation have been inconsistent, the present study provides longitudinal evidence that the P2 evoked by speech sounds remains stable between the ages of 14 and 17. This finding suggests that neural generators of P2 are structurally stable during adolescence and the pathways leading to them are fully myelinated by age 14. Moreover, though it is clear that P2 at least in part reflects contributions from auditory and other cortical regions (Wyss et al., 2014), this interpretation is consistent with the idea that P2 reflects contributions from the reticular activating system (Velasco et al., 1985; Velasco and Velasco, 1986; Crowley and Colrain, 2004), a subcortical network of nuclei involved with arousal state regulation that is thought to be fully developed by early childhood (Ponton et al., 2000).

#### N2

We observed a reduction in N2 latency across adolescence, consistent with cross-sectional findings for N2 elicited by both tone and speech stimuli under passive listening conditions (Martin et al., 1988; Tonnquist-Uhlén, 1996; Cunningham et al., 2000; Sussman et al., 2008). N2 is thought to be a composite measure with contributions from cortical, thalamic, and brainstem generators (Velasco et al., 1985; Velasco and Velasco, 1986; Ponton et al., 2002; Mahajan and McArthur, 2012); the observed reduction in N2 latency suggests that myelination of white matter tracts connecting these generators increases during adolescence. Further, the observed reductions in peak latency only for CAEP components thought to rely more heavily on cortico-cortical signaling (N1, N2) suggests that these reductions are driven by increased myelination of cortico-cortical pathways during adolescence. Alternatively, the N2 latency decrease could reflect a shift in the relative contributions of the multiple neural generators of N2, such that an earlier N2 subcomponent becomes the largest contributor to overall N2 amplitude with age, driving the apparent peak latency earlier. Such a pattern could occur as a result of synaptic pruning over different timescales across N2 neural generators. It should be noted that in the present study, the neural generators of the broad N2 component may be particularly heterogenous as they may also include neural regions underlying sound offset processing due to stimulus length (170 ms). While increased myelination in cortico-cortical pathways with age is a more parsimonious, and perhaps more likely, explanation for the reduced N2 latency observed in adolescence, future work tracking the developmental trajectory of N2 subcomponents independently in this age range is necessary to arbitrate these interpretations.

We observed a reduction in N2 amplitude during adolescence, which is consistent with the majority of previous cross-sectional work (Enoki et al., 1993; Johnstone et al., 1996; Oades et al., 1997; Cunningham et al., 2000; Ponton et al., 2000; Bishop et al., 2007; Sussman et al., 2008; Mahajan and McArthur, 2012). This reduction suggests an increased efficiency in the neural generators of N2 during adolescence. The increased efficiency may reflect functional reorganization of the circuits underlying N2 related to an adolescent increase in inhibitory control (e.g., van der Stelt et al., 1998; van der Molen, 2000). Neurobehavioral relationships demonstrate that increases in N2 amplitude reflect online inhibitory processes (Kopp et al., 1996), and that the increase in N2 amplitude is larger when inhibition requires greater effort (Kopp et al., 1996). Conversely, better performance on inhibitory executive control tasks, which presumably indicates more efficient inhibitory cognitive mechanisms, is associated with a smaller increase in N2 amplitude during online inhibition (Lamm et al., 2006). The reduced N2 amplitude observed in the present study may therefore reflect a reduction in effort required to suppress an irrelevant sound stimulus during adolescence. However, previous associations of N2 amplitude with online inhibition have measured N2 elicited by visual stimuli during active inhibition tasks; future work examining whether the amplitude of N2 elicited by auditory stimuli is associated with sound inhibition difficulty is needed to test this hypothesis.

### Maturation of CAEP Variability

We observed a decrease in CAEP variability between the ages of 14 and 17, indicating that the passive cortical response to sound becomes more stable across adolescence. Notably, unlike the observed changes in latency and amplitude, the decrease in CAEP variability with age was present and similar across all components. It is also interesting to note that the observed decrease in CAEP variability is opposite in direction to the increase observed for brainstem AEP variability during adolescence (Skoe et al., 2015; Krizman et al., 2015). This suggests that the stability of sound encoding is not uniform across the auditory system, and that the relative stability of encoding among regions may be an important index of auditory system maturity.

We suggest that this decrease in variability reflects stabilizing of trial-to-trial CAEP amplitude and latency during adolescence as a function of synaptic pruning and myelination. As unnecessary synapses are eliminated and the auditory circuits underlying CAEP generation are refined, activity within those circuits should become more constrained to only neurons integral to CAEP generation. Across repeated activations of the circuit the set of neurons activated should then be more homogenous, leading to less variable CAEP amplitude at the scalp. Additionally, increased myelination in the auditory system could reduce the trial-to-trial variability of CAEP component timing by reducing component latency to the lowest value possible on every response, reducing temporal jitter and leading to decreased amplitude variability across averaged responses. Future work examining the relationship between CAEP component variabilities and intertrial phase coherence during adolescence could help distinguish between circuit refinement and timing stabilization interpretations of the observed variability decrease. Alternatively, it is possible that the decrease in CAEP variability is driven by reductions in oscillatory neural activity that is not phase-locked to sound onset, which would be less apparent in latency and amplitude measures taken on the averaged CAEP responses. The observation of the variability decrease during the prestimulus period and the similarity of the decrease across time windows supports this interpretation, and decreases in free-running neural oscillations within multiple frequency bands have been previously reported during adolescence (Gasser et al., 1988; Tierney et al., 2014) and associated with synaptic pruning (Whitford et al., 2007). If decreases in oscillatory neural activity underlie the adolescent reduction in CAEP variability, the variability decrease may or may not represent neural activity directly related to processing an auditory event. Future work examining maturation of auditory event-related spectral perturbations (Makeig, 1993) could clarify this issue by determining whether adolescent decreases in oscillatory EEG power change in an event-related manner.

## Conclusion

Our results provide longitudinal evidence that the CAEP continues to mature through adolescence; these functional changes are interpreted as the products of ongoing structural change in the auditory system during this time period. Decreases in N1 and N2 latency between ages 14 and 17 suggest that myelination in cortico-cortical white matter pathways continues through adolescence, whereas the absence of concurrent latency changes for P1 or P2 suggests that myelination of thalamo-cortical pathways is stable during this time period and myelination is complete in the reticular activating system by age 14. Decreases in P1 and N2 amplitude between ages 14 and 17 indicate increased neural efficiency in the generators of these components with age, whereas unchanging P2 amplitude indicates the neural generators of P2 are stable during adolescence. Increasing N1 amplitude between ages 15 and 17 suggests functional reorganization of auditory circuits underlying N1 during adolescence. Decreases in CAEP variability indicate that the basic response to sound becomes more stable across adolescence. Taken together, these findings indicate that multiple aspects of cortical auditory function continue to mature during the transition from childhood to adulthood. Importantly, this continued maturation of function suggests that the neural generators of the CAEP remain plastic during adolescence, and potentially amenable to enhancement, deprivation, or remediation with experience during this time. While the present work describes CAEP maturation in a general adolescent population with a broad range of auditory experience, future work can investigate how adolescent CAEP maturation is affected by specific types of experience known to influence the auditory system, including musical training, linguistic experience, and environmental conditions.

## Conflict of Interest Statement

The authors declare that the research was conducted in the absence of any commercial or financial relationships that could be construed as a potential conflict of interest.

## References

[B1] Albrecht R. von Suchodoletz W. Uwer R. (2000). The development of auditory evoked dipole source activity from childhood to adulthood. Clin. Neurophysiol. 111, 2268–2276. 10.1016/s1388-2457(00)00464-8 11090781

[B2] Bishop D. V. M. Hardiman M. Uwer R. von Suchodoletz W. (2007). Maturation of the long-latency auditory ERP: step function changes at start and end of adolescence. Dev. Sci. 10, 565–575. 10.1111/j.1467-7687.2007.00619.x 17683343 PMC2121131

[B3] Buschman T. J. Miller E. K. (2007). Top-down versus bottom-up control of attention in the prefrontal and posterior parietal cortices. Science 315, 1860–1862. 10.1126/science.1138071 17395832

[B4] Crowley K. E. Colrain I. M. (2004). A review of the evidence for P2 being an independent component process: age, sleep and modality. Clin. Neurophysiol. 115, 732–744. 10.1016/j.clinph.2003.11.021 15003751

[B5] Cunningham J. Nicol T. Zecker S. Kraus N. (2000). Speech-evoked neurophysiologic responses in children with learning problems: development and behavioral correlates of perception. Ear Hear. 21, 554–568. 10.1097/00003446-200012000-00003 11132782

[B6] Delorme A. Makeig S. (2004). EEGLAB: an open source toolbox for analysis of single-trial EEG dynamics including independent component analysis. J. Neurosci. Methods 134, 9–21. 10.1016/j.jneumeth.2003.10.009 15102499

[B7] Eggermont J. J. Moore J. K. (2012). “Morphological and functional development of the auditory nervous system,” in Human Auditory Development Springer Handbook of Auditory Research, eds Werner L. Fay R. R. Popper A. N. (New York, NY: Springer), 61–105.

[B8] Enoki H. Sanada S. Yoshinaga H. Oka E. Ohtahara S. (1993). The effects of age on the N200 component of the auditory event-related potentials. Brain Res. Cogn. Brain Res. 1, 161–167. 10.1016/0926-6410(93)90023-x 8257871

[B9] Gasser T. Verleger R. Bächer P. Sroka L. (1988). Development of the EEG of school-age children and adolescents. I. Analysis of band power. Electroencephalogr. Clin. Neurophysiol. 69, 91–99. 10.1016/0013-4694(88)90204-0 2446839

[B10] Giedd J. N. Blumenthal J. Jeffries N. O. Castellanos F. X. Liu H. Zijdenbos A.. (1999). Brain development during childhood and adolescence: a longitudinal MRI study. Nat. Neurosci. 2, 861–863. 10.1038/13158 10491603

[B11] Gogtay N. Giedd J. N. Lusk L. Hayashi K. M. Greenstein D. Vaituzis A. C.. (2004). Dynamic mapping of human cortical development during childhood through early adulthood. Proc. Natl. Acad. Sci. U S A 101, 8174–8179. 10.1073/pnas.0402680101 15148381 PMC419576

[B12] Goodin D. S. Squires K. C. Henderson B. H. Starr A. (1978). Age-related variations in evoked potentials to auditory stimuli in normal human subjects. Electroencephalogr. Clin. Neurophysiol. 44, 447–458. 10.1016/0013-4694(78)90029-9 76553

[B13] Hecox K. Galambos R. (1974). Brain stem auditory evoked responses in human infants and adults. Arch. Otolaryngol. 99, 30–33. 10.1001/archotol.1974.00780030034006 4809084

[B14] Huttenlocher P. R. (1979). Synaptic density in human frontal cortex—Developmental changes and effects of aging. Brain Res. 163, 195–205. 10.1016/0006-8993(79)90349-4 427544

[B15] Johnson R. Jr. (1989). Developmental evidence for modality-dependent P300 generators: a normative study. Psychophysiology 26, 651–667. 10.1111/j.1469-8986.1989.tb03167.x 2629013

[B16] Johnstone S. J. Barry R. J. Anderson J. W. Coyle S. F. (1996). Age-related changes in child and adolescent event-related potential component morphology, amplitude and latency to standard and target stimuli in an auditory oddball task. Int. J. Psychophysiol. 24, 223–238. 10.1016/s0167-8760(96)00065-7 8993997

[B17] Klatt D. H. (1980). Software for a cascade/parallel formant synthesizer. J. Acoust. Soc. Am. 67, 971–995. 10.1121/1.383940

[B18] Kopp B. Rist F. Mattler U. (1996). N200 in the flanker task as a neurobehavioral tool for investigating executive control. Psychophysiology 33, 282–294. 10.1111/j.1469-8986.1996.tb00425.x 8936397

[B19] Krizman J. Skoe E. Marian V. Kraus N. (2014). Bilingualism increases neural response consistency and attentional control: evidence for sensory and cognitive coupling. Brain Lang. 128, 34–40. 10.1016/j.bandl.2013.11.006 24413593 PMC3923605

[B20] Krizman J. Tierney A. Fitzroy A. B. Skoe E. Amar J. Kraus N. (2015). Continued maturation of auditory brainstem function during adolescence: a longitudinal approach. Clin. Neurophysiol. [Epub ahead of print]. 10.1016/j.clinph.2015.01.026 25801342 PMC4550576

[B21] Lamm C. Zelazo P. D. Lewis M. D. (2006). Neural correlates of cognitive control in childhood and adolescence: disentangling the contributions of age and executive function. Neuropsychologia 44, 2139–2148. 10.1016/j.neuropsychologia.2005.10.013 16310813

[B22] Lawrence M. A. (2013). Ez: Easy analysis and visualization of factorial experiments. Available online at: http://CRAN.R-project.org/package=ez

[B23] Liégeois-Chauvel C. Musolino A. Badier J. M. Marquis P. Chauvel P. (1994). Evoked potentials recorded from the auditory cortex in man: evaluation and topography of the middle latency components. Electroencephalogr. Clin. Neurophysiol. 92, 204–214. 10.1016/0168-5597(94)90064-7 7514990

[B24] Lopez-Calderon J. Luck S. J. (2014). ERPLAB: an open-source toolbox for the analysis of event-related potentials. Front. Hum. Neurosci. 8:213. 10.3389/fnhum.2014.00213 24782741 PMC3995046

[B25] Mahajan Y. McArthur G. (2012). Maturation of auditory event-related potentials across adolescence. Hear. Res. 294, 82–94. 10.1016/j.heares.2012.10.005 23103362

[B26] Makeig S. (1993). Auditory event-related dynamics of the EEG spectrum and effects of exposure to tones. Electroencephalogr. Clin. Neurophysiol. 86, 283–293. 10.1016/0013-4694(93)90110-h 7682932

[B27] Mäkelä J. P. Hari R. (1992). Neuromagnetic auditory evoked responses after a stroke in the right temporal lobe. Neuroreport 3, 94–96. 10.1097/00001756-199201000-00025 1611040

[B28] Martin L. Barajas J. J. Fernandez R. Torres E. (1988). Auditory event-related potentials in well-characterized groups of children. Electroencephalogr. Clin. Neurophysiol. 71, 375–381. 10.1016/0168-5597(88)90040-8 2457489

[B29] McArthur G. C. Bishop D. (2002). Event-related potentials reflect individual differences in age-invariant auditory skills. Neuroreport 13, 1079–1082. 10.1097/00001756-200206120-00021 12060813

[B30] Moore J. K. Guan Y.-L. (2001). Cytoarchitectural and axonal maturation in human auditory cortex. J. Assoc. Res. Otolaryngol. 2, 297–311. 10.1007/s101620010052 11833605 PMC3201070

[B31] Näätänen R. Picton T. W. (1987). The N1 wave of the human electric and magnetic response to sound: a review and an analysis of the component structure. Psychophysiology 24, 375–425. 10.1111/j.1469-8986.1987.tb00311.x 3615753

[B32] Oades R. D. Dittmann-Balcar A. Zerbin D. (1997). Development and topography of auditory event-related potentials (ERPs): mismatch and processing negativity in individuals 8–22 years of age. Psychophysiology 34, 677–693. 10.1111/j.1469-8986.1997.tb02143.x 9401422

[B33] Pang E. W. Taylor M. J. (2000). Tracking the development of the N1 from age 3 to adulthood: an examination of speech and non-speech stimuli. Clin. Neurophysiol. 111, 388–397. 10.1016/s1388-2457(99)00259-x 10699397

[B34] Parbery-Clark A. Skoe E. Kraus N. (2009). Musical experience limits the degradative effects of background noise on the neural processing of sound. J. Neurosci. 29, 14100–14107. 10.1523/JNEUROSCI.3256-09.2009 19906958 PMC6665054

[B35] Paus T. (2005). Mapping brain maturation and cognitive development during adolescence. Trends Cogn. Sci. 9, 60–68. 10.1016/j.tics.2004.12.008 15668098

[B36] Paus T. Collins D. L. Evans A. C. Leonard G. Pike B. Zijdenbos A. (2001). Maturation of white matter in the human brain: a review of magnetic resonance studies. Brain Res. Bull. 54, 255–266. 10.1016/s0361-9230(00)00434-2 11287130

[B37] Paus T. Zijdenbos A. Worsley K. Collins D. L. Blumenthal J. Giedd J. N.. (1999). Structural maturation of neural pathways in children and adolescents: *in vivo* study. Science 283, 1908–1911. 10.1126/science.283.5409.1908 10082463

[B38] Picton T. W. Alain C. Woods D. L. John M. S. Scherg M. Valdes-Sosa P.. (1999). Intracerebral sources of human auditory-evoked potentials. Audiol. Neurootol. 4, 64–79. 10.1159/000013823 9892757

[B39] Ponton C. W. Eggermont J. J. (2001). Of kittens and kids: altered cortical maturation following profound deafness and cochlear implant use. Audiol. Neurootol. 6, 363–380. 10.1159/000046846 11847464

[B40] Ponton C. W. Eggermont J. J. Khosla D. Kwong B. Don M. (2002). Maturation of human central auditory system activity: separating auditory evoked potentials by dipole source modeling. Clin. Neurophysiol. 113, 407–420. 10.1016/s1388-2457(01)00733-7 11897541

[B41] Ponton C. W. Eggermont J. J. Kwong B. Don M. (2000). Maturation of human central auditory system activity: evidence from multi-channel evoked potentials. Clin. Neurophysiol. 111, 220–236. 10.1016/s1388-2457(99)00236-9 10680557

[B42] Posner M. I. Walker J. A. Friedrich F. J. Rafal R. D. (1984). Effects of parietal injury on covert orienting of attention. J. Neurosci. 4, 1863–1874. 6737043 10.1523/JNEUROSCI.04-07-01863.1984 PMC6564871

[B43] R Core Team (2014). R: A Language and Environment for Statistical Computing. Vienna: R Foundation for Statistical Computing.

[B44] Reiss A. L. Abrams M. T. Singer H. S. Ross J. L. Denckla M. B. (1996). Brain development, gender and IQ in children. Brain 119, 1763–1774. 10.1093/brain/119.5.1763 8931596

[B45] R Studio Team (2014). R Studio: Integrated Development for R. Boston, MA: R Studio, Inc.

[B46] Scarff C. J. Reynolds A. Goodyear B. G. Ponton C. W. Dort J. C. Eggermont J. J. (2004). Simultaneous 3-T fMRI and high-density recording of human auditory evoked potentials. Neuroimage 23, 1129–1142. 10.1016/j.neuroimage.2004.07.035 15528112

[B47] Scherg M. Von Cramon D. (1985). Two bilateral sources of the late AEP as identified by a spatio-temporal dipole model. Electroencephalogr. Clin. Neurophysiol. 62, 32–44. 10.1016/0168-5597(85)90033-4 2578376

[B48] Sharma A. Kraus N. McGee T. J. Nicol T. G. (1997). Developmental changes in P1 and N1 central auditory responses elicited by consonant-vowel syllables. Electroencephalogr. Clin. Neurophysiol. 104, 540–545. 10.1016/s0168-5597(97)00050-6 9402896

[B49] Shaw N. A. (1988). The auditory evoked potential in the rat—a review. Prog. Neurobiol. 31, 19–45. 10.1016/0301-0082(88)90021-4 3287454

[B50] Skoe E. Krizman J. Anderson S. Kraus N. (2015). Stability and plasticity of auditory brainstem function across the lifespan. Cereb. Cortex 25, 1415–1426. 10.1093/cercor/bht311 24366906 PMC4428291

[B51] Strait D. L. Kraus N. (2011). Can you hear me now? Musical training shapes functional brain networks for selective auditory attention and hearing speech in noise. Front. Psychol. 2:113. 10.3389/fpsyg.2011.00113 21716636 PMC3115514

[B52] Strait D. L. Slater J. Abecassis V. Kraus N. (2014). Cortical response variability as a developmental index of selective auditory attention. Dev. Sci. 17, 175–186. 10.1111/desc.12107 24267508 PMC4119172

[B53] Sussman E. Steinschneider M. Gumenyuk V. Grushko J. Lawson K. (2008). The maturation of human evoked brain potentials to sounds presented at different stimulus rates. Hear. Res. 236, 61–79. 10.1016/j.heares.2007.12.001 18207681 PMC2567844

[B54] Tierney A. Strait D. L. Kraus N. (2014). Resting gamma power is linked to reading ability in adolescents. Dev. Sci. 17, 86–93. 10.1111/desc.12094 24341975

[B55] Tonnquist-Uhlén I. (1996). Topography of auditory evoked long-latency potentials in children with severe language impairment: the P2 and N2 components. Ear Hear. 17, 314–326. 10.1097/00003446-199608000-00003 8862969

[B56] van der Molen M. W. (2000). Developmental changes in inhibitory processing: evidence from psychophysiological measures. Biol. Psychol. 54, 207–239. 10.1016/s0301-0511(00)00057-0 11035224

[B57] van der Stelt O. Kok A. Smulders F. T. Y. Snel J. Boudewijn Gunning W. (1998). Cerebral event-related potentials associated with selective attention to color: developmental changes from childhood to adulthood. Psychophysiology 35, 227–239. 10.1111/1469-8986.3530227 9564743

[B58] Vaughan H. G. Jr. Ritter W. (1970). The sources of auditory evoked responses recorded from the human scalp. Electroencephalogr. Clin. Neurophysiol. 28, 360–367. 10.1016/0013-4694(70)90228-2 4191187

[B59] Velasco M. Velasco F. (1986). Subcortical correlates of the somatic, auditory and visual vertex activities. II. Referential EEG responses. Electroencephalogr. Clin. Neurophysiol. 63, 62–67. 10.1016/0013-4694(86)90063-5 2416537

[B60] Velasco M. Velasco F. Olvera A. (1985). Subcortical correlates of the somatic, auditory and visual vertex activities in man. I. Bipolar EEG responses and electrical stimulation. Electroencephalogr. Clin. Neurophysiol. 61, 519–529. 10.1016/0013-4694(85)90971-x 2415327

[B61] Wechsler D. (1999). Wechsler Abbreviated Scale of Intelligence. San Antonio, TX: The Psychological Corporation (Harcourt Brace and Company).

[B62] Whitford T. J. Rennie C. J. Grieve S. M. Clark C. R. Gordon E. Williams L. M. (2007). Brain maturation in adolescence: concurrent changes in neuroanatomy and neurophysiology. Hum. Brain Mapp. 28, 228–237. 10.1002/hbm.20273 16767769 PMC6871488

[B63] Wickham H. (2007). Reshaping data with the reshape package. J. Stat. Softw. 21, 1–20.

[B64] Wyss C. Boers F. Kawohl W. Arrubla J. Vahedipour K. Dammers J.. (2014). Spatiotemporal properties of auditory intensity processing in multisensor MEG. Neuroimage 102, 465–473. 10.1016/j.neuroimage.2014.08.012 25132019

